# Porcine rotavirus C in pigs with gastroenteritis on Thai swine farms, 2011–2016

**DOI:** 10.7717/peerj.4724

**Published:** 2018-05-08

**Authors:** Supansa Tuanthap, Cherdpong Phupolphan, Supol Luengyosluechakul, Ausanee Duang-in, Apiradee Theamboonlers, Suphot Wattanaphansak, Sompong Vongpunsawad, Alongkorn Amonsin, Yong Poovorawan

**Affiliations:** 1 Inter-Department Program of Biomedical Sciences, Faculty of Graduate School, Chulalongkorn University, Bangkok, Thailand; 2 The Livestock Animal Hospital, Faculty of Veterinary Science, Chulalongkorn University, Nakorn Pathom, Thailand; 3 Department of Veterinary Medicine, Faculty of Veterinary Science, Chulalongkorn University, Bangkok, Thailand; 4 Center of Excellence in Clinical Virology, Faculty of Medicine, Chulalongkorn University, Bangkok, Thailand; 5 Department of Veterinary Public Health, Faculty of Veterinary Science, Chulalongkorn University, Bangkok, Thailand

**Keywords:** Thailand, VP7, VP4, Pigs, Rotavirus C

## Abstract

Swine are economically important food animals, but highly contagious porcine epidemic diarrhea virus (PEDV) and rotavirus can afflict pig herds and contribute significantly to piglet morbidity and mortality. While there have been studies on rotavirus group A (RVA) in Thailand, reports of rotavirus group C (RVC) are limited. Here, we aimed to identify the prevalence of RVC circulating on Thai commercial swine farms. We analyzed 769 feces and intestine mucosal contents of pigs affected with diarrhea between 2011 and 2016 using RT-PCR specific for the PEDV spike (S), rotavirus glycoprotein (G) VP7, and protease-sensitive protein (P) VP4 genes. We found that 6.6% (51/769) of samples tested positive for RVC, of which 11 samples were co-infected with RVA and four samples were co-infected with PEDV. Three samples tested positive for all three viruses. Phylogenetic analysis of the VP7 gene showed that the most frequent RVC genotype was G1, which grouped with the prototypic RVC Cowden strain. While G6 and G9 were also common, G3 was relatively rare. Analysis of the VP4 gene revealed that the most common P type was P[5], followed by P[4], P[7], and P[1]. In all, there were six G/P combinations (G6P[5], G1P[1], G1P[4], G1P[5], G9P[4], and G9P[7]), of which G6P[5] was the most predominant.

## Introduction

Pork production is an important economic output for many countries including Thailand. Despite stringent good husbandry practices, diseases affecting the pig herd such as diarrhea are not uncommon especially in high-density commercial farms. Diarrhea is associated with high morbidity and mortality rates in suckling and post-weaning piglets. Frequent viral etiologies are rotavirus and porcine epidemic diarrhea virus (PEDV). Infections generally occur via fecal-oral route and result in significant economic impact due to animal loss, sanitation efforts, and reduced pork production. Without molecular diagnostics, PEDV, and rotavirus infections are difficult to ascertain and differentiate as they cause similar clinical symptoms and frequently co-infect pigs. In addition, rotavirus zoonosis in pigs can sometimes lead to infection in humans, especially among farm workers who have close-contact with potentially infected animals.

Enteric virus replication blunts the villous enterocytes in the intestine, resulting in electrolyte imbalance, intestinal malabsorption, watery diarrhea, dehydration, and often death (Jung et al., 2015; Chang et al., 1999). Pigs of all ages are susceptible to these viral infections, which can manifest in different disease severity depending on the age of the animal (Ciarlet et al., 2002; Neog et al., 2011; Pott et al., 2012; Riepenhoff-Talty et al., 1982). Neonatal and post-weaned piglets are most vulnerable due to the lack of protective immunity (Bohl et al., 1982). Asymptomatic infection in adult pigs further complicate efforts to identify and quarantine sick animals, which are crucial in preventing the spread of infection (Collins, Martella & O’Shea, 2008; Marthaler et al., 2013; Saif et al., 1980; Theuns et al., 2016; Zhou et al., 2016).

Rotavirus is a member of the family *Reoviridae*, genus *Rotavirus*. Viral particles are icosahedral and non-enveloped, with concentric triple-layer capsid protein shell comprising of the viral proteins VP7, VP6, and VP2. The genome is comprised of 11 double-stranded RNA segments. Among the most common to infect swine, rotavirus group A (RVA) affects piglets between one and three weeks of age, while rotavirus group C (RVC) frequently causes diarrhea in pre- and post-weaning piglets (Gouvea et al., 1991; Martella et al., 2007; Marthaler et al., 2013). PEDV is a member of the family *Coronaviridae* in the genus *Alphacoronavirus*. It is an enveloped virus with positive-sense, single-stranded RNA genome of approximately 28 kb. The spike (S) and ORF3 gene sequences generally display the most genetic diversity and are used in differentiating strains. Immunity after PEDV infection is not life-long and vaccinations have historically been ineffective.

Molecular characterization of rotavirus relies on the binary classification using VP7 or glycoprotein (G) and VP4 or protease-sensitive protein (P). Among RVA associated with infection in pigs, there are 12 G genotypes (G1–G6, G8–G12, and G26) and 16 P genotypes (P[1] to P[8], P[11], P[13], P[19], P[23], P[26], P[27], P[32], and P[34]) (Vlasova, Amimo & Saif, 2017). For RVC, there are currently nine G genotypes and seven P genotypes. This genetic diversity renders most pig herds susceptible to repeated RVA and RVC infection. Thus, awareness of the circulating porcine RVC on pig farms is critical in evaluating the disease burden and the potential impact of widespread infection. RVC infection is currently not well-studied in Thailand due to the lack of disease awareness, vaccine availability, and access to molecular diagnostics. The objective of this study is to investigate the prevalence and to characterize RVC found in pigs with diarrhea on commercial farms in Thailand.

## Materials and Methods

### Specimen collection and preparation

The Institutional Animal Care and Use Committee (IACUC number 1731020) and the Institutional Biosafety Committee (IBC number 1731008) of Chulalongkorn University approved this study. Feces and small intestine contents from pigs of various ages with watery diarrhea were submitted to the Livestock Animal Hospital at the Chulalongkorn University Faculty of Veterinary Science in Nakorn Pathom province between May 2011 and August 2016 for viral testing. There were 769 samples from 2011 (*n* = 40), 2012 (*n* = 95), 2013 (*n* = 87), 2014 (*n* = 158), 2015 (*n* = 164), and 2016 (*n* = 225). These represent archived and convenient samples from 123 commercial pig farms located throughout Thailand, of which 316 were from western provinces (Kanchanaburi, Prachuap Khiri Khan, Phetchaburi, and Ratchaburi), 173 were from central provinces (Lop Buri, Samut Songkhram, Suphan Buri, Saraburi, Phra Nakhon Si Ayutthaya, and Nakhon Pathom), 109 were from eastern provinces (Chon Buri and Chachoengsao), 80 were from northeastern provinces (Ubon Ratchathani, Udon Thani, and Nakhon Ratchasima), 26 were from southern provinces (Trang and Nakhon Si Thammarat), and 65 were from unspecified locations (Table S1). Samples were categorized into the following age groups: 0–6 days, 1–4 weeks (pre-weaning), ≥4–8 weeks (early nursery), ≥8–12 weeks (late nursery), >12 weeks (starter–finisher), and sow (both pregnant and lactating).

The intestine mucosa were collected from dead animals by scraping the duodenum and upper part of the jejunum, particularly the thin walled area where gas accumulated inside the lumen. Approximately 10% (v/v) of mucosal or fecal suspensions in sterile phosphate-buffered saline (0.1 M, pH 7.2) were centrifuged at 3,000*g* for 20 min and the supernatants collected.

### Viral nucleic acid detection

Nucleic acid was extracted using Ribospin vRD II viral RNA extraction kit (GeneAll, Seoul, Korea) according to the manufacturer’s instructions. The partial S gene of PEDV, VP7 gene of RVA/RVC, and VP4 gene of RVC were amplified using SuperScript III One-Step RT-PCR System with Platinum *Taq* DNA polymerase (Invitrogen, Carlsbad, CA, USA). Samples were reverse-transcribed at 48 °C for 45 min. Cycling parameters were initial denaturation at 95 °C for 2 min, followed by 35 cycles at 94 °C for 30 s, 52 °C or 55 °C for 1 min, 72 °C for 90 s, and final extension at 72 °C for 5 min. Primer sequences are shown in Table 1. Amplicons were purified using agarose gel electrophoresis and sequenced. Nucleotide (nt) sequences were deposited in the GenBank database under the accession numbers KX911667–KX911708, MF139507–MF139509 and MF139516–MF139517 (VP7) and MG575522–MG575532 (VP4).

**Table 1:** Oligonucleotide primers used in this study.

Primers	Nucleotide sequence (5′ to 3′)	Position	Annealing temperature	Product size
**PEDV S gene** (Kim, Song & Park, 2001)	TTCTGAGTCACGAACAGCCA	1466–1485	55 °C	651 bp
	CATATGCAGCCTGCTCTGAA	2097–2116		
**RVA VP7 gene** (accession number AB176677.1)	VP7-CU-RVAF: CGGTTAGCTCCTTTTAATGT	33–52	55 °C	891 bp
	VP7-CU-RVAR: CATTTCTTCCAATTTACTCGC	903–924		
**RVC VP7 gene** (accession number M61101.1)	VP7-CU-RVCF: GAAGCTGTCTGACAAACTGG	17–36	52 °C	1,046 bp
	VP7-CU-RVCR: GCCACATGATCTTGTTTACGC	1042–1061		
**RVC VP4 gene** (Diaz-Salinas et al., 2013)	VP4-17Fdeg: GATCRATGGCGTCYTCAC	17–34	55 °C	1,222 bp
	VP4-1238R: CCTGATGAATGTAATCCWGGAT	1216–1238		

### Analysis of the RVC VP4 and VP7 genes

Sequences were assembled using SeqMan sequence analysis software version 6 (DNASTAR) and aligned using Clustral X version 2.0.11 (Larkin et al., 2007). Phylogenetic trees were reconstructed with reference sequences available in the GenBank database using the maximum-likelihood method and 1,000 pseudo-replicates implemented in MEGA6 software (Tamura et al., 2013). Bootstrap values >85% were considered significant for the VP7 gene and >80% for the VP4 gene. Prototypic RVC strain Cowden (G1P[1]), Shintoku (G2P[3]), HF (G3, undetermined P) and Bristol (G4P[2]) served as reference strains.

## Results

### Viral detection

Between 2011 and 2016, 19.9% (153/769) of the samples tested positive for PEDV. The overwhelming majority of the samples were from 0 to 6 day-old piglets (Fig. S1). RVA was found in 9.5% (73/769) of the samples, while RVC was identified in 6.6% (51/769) of the samples. One-fifth of the samples (21.6%, 11/51) were co-infected with RVA/RVC, most of which were from piglets ≥4–8 weeks of age. Fewer PEDV-positive samples were co-infected with RVA (1.8%, 14/769) than RVC (7.8%, 4/51). Only three samples tested positive for all three viruses.

### Sequence and phylogenetic analysis of the RVC VP7 gene

**Figure 1:**
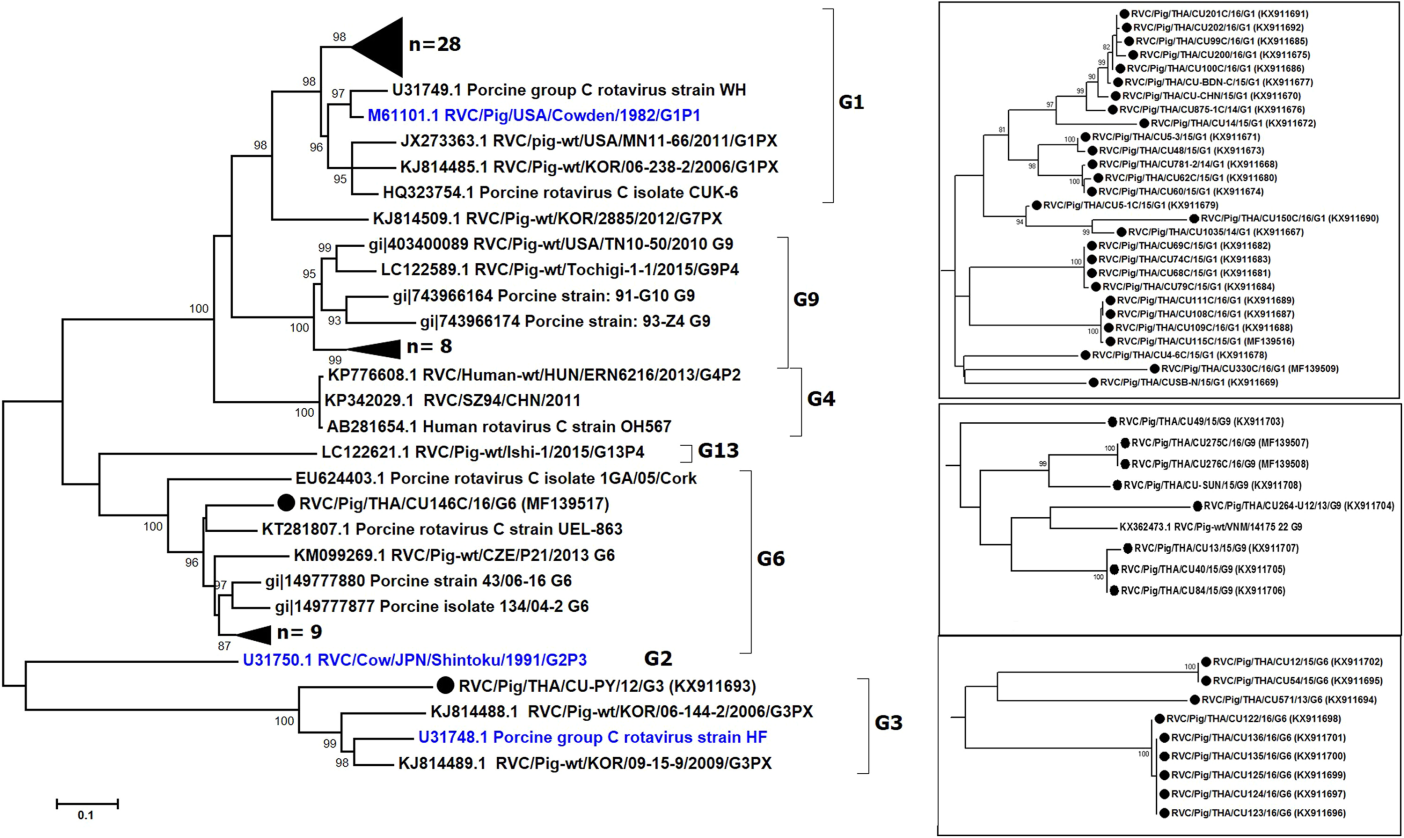
Phylogenetic analysis of the RVC VP7 gene. Trees were reconstructed with reference sequences available in the GenBank database using the maximum-likelihood method and 1,000 pseudo-replicates implemented in MEGA6. Bootstrap values >85% were considered significant. Strains identified in this study are shown as triangles (or dotted in the inset). RVC reference strains are blue.

We sought to focus our study on RVC and therefore examined the G and P genotypes. Sufficient sequences of VP7 were successfully obtained from 47 samples, most of which were derived from feces (Table 2). The near full-length VP7 sequences were compared to the RVC references available in the GenBank database. Phylogenetic analysis showed that the RVC in this study belonged to G1 (55%, 28/51), G3 (2%, 1/51), G6 (20%, 10/51), and G9 (16%, 8/51) (Fig. 1). The G1 strains were closely related to the prototypic Cowden (86.1–91.7% nt identity). The lone G3 strain RVC/Pig/THA/CU-PY/12/G3 was distantly related to the prototypic HF (78%). The G6 strains shared high identity to a porcine rotavirus strain ITA/43/06-16 isolated in Italy in 2005 (88.6–90.9% nt identity) and the G9 strains were closely related to a Vietnamese porcine rotavirus strain (strain RVC/Pig-wt/VNM/14175_22) (86.3–89.5% nt identity).

**Table 2:** The 47 RVC strains with sequences from this study.

Collection year	Strain name	Age of host (week)	Sample	RVC genotype		RVA	PEDV
				VP7	VP4		
**2012**	RVC/Pig/THA/CU-PY/12/G3	1–4	Small intestine	G3			
**2013**	RVC/Pig/THA/CU571/13/G6	n/a	Feces	G6			
	RVC/Pig/THA/CU264-U12/13/G9	n/a	Feces	G9	P[7]		
**2014**	RVC/Pig/THA/CU875-1C/14/G1	5–8	Small intestine	G1		**+**	
	RVC/Pig/THA/CU1035/14/G1	1–4	Feces	G1			**+**
	RVC/Pig/THA/CU781-2/14/G1	1–4	Small intestine	G1			
**2015**	RVC/Pig/THA/CU-SUN/15/G9	5–8	Feces	G9		**+**	
	RVC/Pig/THA/CU-BDN-C/15/G1	5–8	Feces	G1		**+**	
	RVC/Pig/THA/CUSB-N/15/G1	5–8	Feces	G1		**+**	
	RVC/Pig/THA/CU-CHN/15/G1	5–8	Feces	G1			
	RVC/Pig/THA/CU4-6C/15/G1	5–8	Small intestine	G1			
	RVC/Pig/THA/CU5-1C/15/G1	5–8	Small intestine	G1			
	RVC/Pig/THA/CU5-3/15/G1	5–8	Small intestine	G1			
	RVC/Pig/THA/CU12/15/G6	5–8	Feces	G6			
	RVC/Pig/THA/CU13/15/G9	1–4	Feces	G9			
	RVC/Pig/THA/CU14/15/G1	5–8	Feces	G1			
	RVC/Pig/THA/CU40/15/G9	5–8	Feces	G9	P[4]	**+**	
	RVC/Pig/THA/CU48/15/G1	5–8	Feces	G1	P[4]		
	RVC/Pig/THA/CU49/15/G9	1–4	Feces	G9			
	RVC/Pig/THA/CU54/15/G6	5–8	Small intestine	G6			
	RVC/Pig/THA/CU60/15/G1	5–8	Small intestine	G1	P[5]		**+**
	RVC/Pig/THA/CU62C/15/G1	5–8	Small intestine	G1			
	RVC/Pig/THA/CU68C/15/G1	5–8	Small intestine	G1			
	RVC/Pig/THA/CU69C/15/G1	5–8	Small intestine	G1			
	RVC/Pig/THA/CU74C/15/G1	1–4	Small intestine	G1			**+**
	RVC/Pig/THA/CU79C/15/G1	0–6 d	Small intestine	G1			**+**
	RVC/Pig/THA/CU84/15/G9	5–8	Feces	G9	P[7]	**+**	**+**
**2016**	RVC/Pig/THA/CU108C/16/G1	5–8	Feces	G1			
	RVC/Pig/THA/CU109C/16/G1	1–4	Feces	G1			
	RVC/Pig/THA/CU111C/16/G1	1–4	Feces	G1			
	RVC/Pig/THA/CU150C/16/G1	5–8	Small intestine	G1			
	RVC/Pig/THA/CU115C/15/G1	5–8	Feces	G1			
	RVC/Pig/THA/CU99C/16/G1	5–8	Feces	G1		**+**	+
	RVC/Pig/THA/CU100C/16/G1	5–8	Feces	G1		+	+
	RVC/Pig/THA/CU122/16/G6	0–6 d	Feces	G6	P[5]		
	RVC/Pig/THA/CU123/16/G6	0–6 d	Feces	G6	P[5]		
	RVC/Pig/THA/CU124/16/G6	0–6 d	Feces	G6	P[5]		
	RVC/Pig/THA/CU125/16/G6	0–6 d	Feces	G6	P[5]		
	RVC/Pig/THA/CU135/16/G6	1–4	Feces	G6	P[5]		
	RVC/Pig/THA/CU136/16/G6	1–4	Feces	G6		**+**	
	RVC/Pig/THA/CU146C/16/G6	5–8	Feces	G6			
	RVC/Pig/THA/CU200/16/G1	5–8	Feces	G1	P[1]	**+**	
	RVC/Pig/THA/CU201C/16/G1	1–4	Feces	G1			
	RVC/Pig/THA/CU202/16/G1	5–8	Feces	G1			
	RVC/Pig/THA/CU275C/16/G9	1–4	Feces	G9			
	RVC/Pig/THA/CU276C/16/G9	1–4	Feces	G9			
	RVC/Pig/THA/CU330C/16/G1	5–8	Feces	G1			

The nearly full-length VP7 sequence encompassing nt 112–952 from the Thai RVC strains encoded amino acid residues 38–316. This region spans the variable region 2 (VR2) to variable region 8 (VR8). Genotype G1 and G9 represented three variable sites at residues 39, 53, and 57 (Table S2). Most G6 strains (9/10 strains) had four residue insertion between amino acid positions 245 and 248 (SSSV/SSTL/SSTM/SSSM) towards the carboxyl terminus of VR8. Potential N-linked glycosylation sites at residues 67–69 and 225–227 and the putative signal cleavage site at residues 49–50 (A/G-Q) were conserved in all the Thai strains in this study.

### Sequence and phylogenetic analysis of the RVC VP4 gene

**Figure 2:**
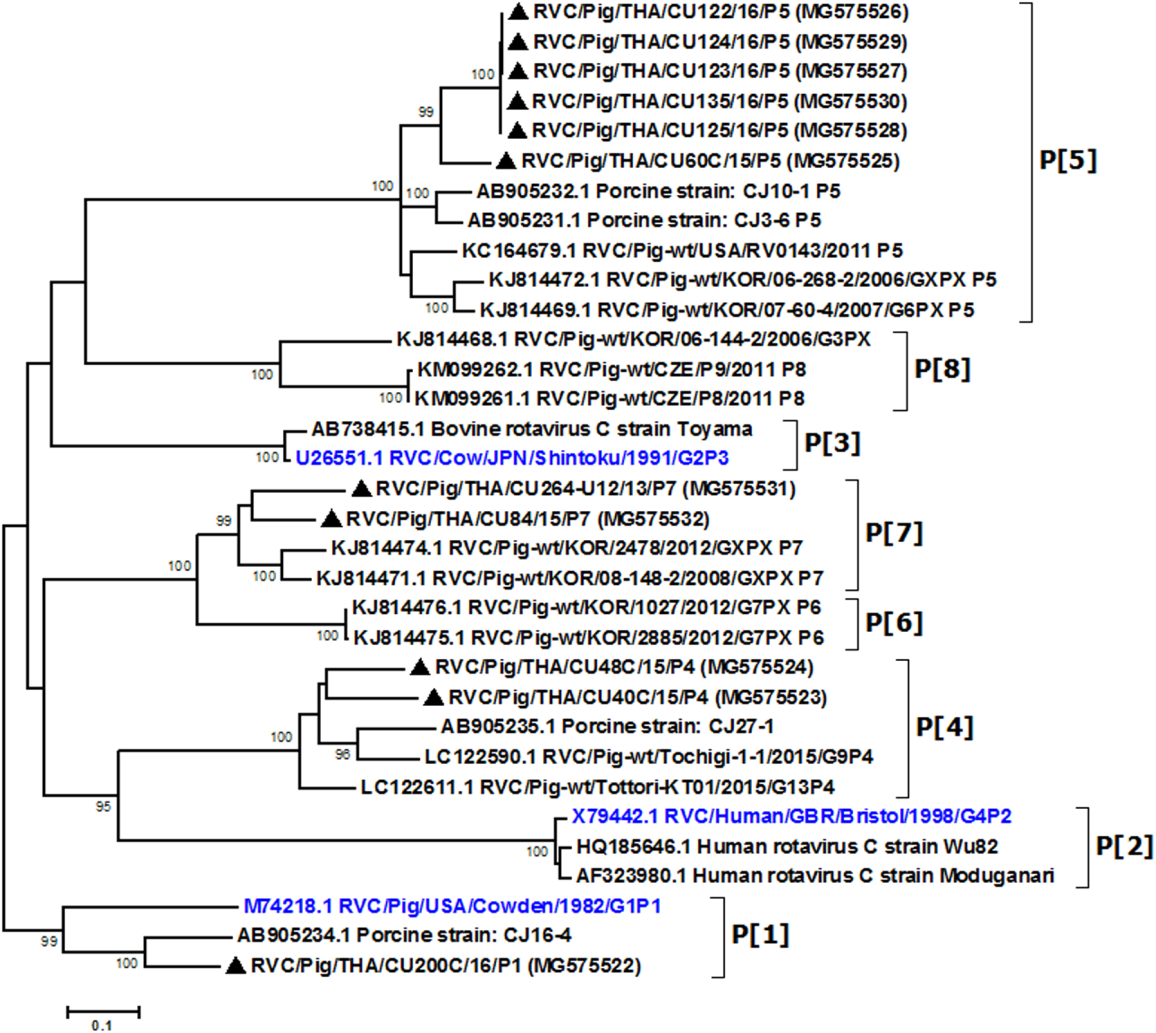
Phylogenetic analysis of the RVC VP4 gene. Trees were reconstructed with reference sequences available in the GenBank database using the maximum-likelihood method and 1,000 pseudo-replicates implemented in MEGA6. Bootstrap values >80% were considered significant. Strains identified in this study are indicated with triangles.

Partial VP4 gene amplification was subsequently performed for all VP7-positive samples. Phylogenetic analysis of the 11 available sequences of VP4 showed that the majority clustered with the genotype P[5] prototype (Fig. 2). Other P genotypes identified were P[1], P[4], and P[7]. In all, there were six G/P combinations (G6P[5], G1P[1], G1P[4], G1P[5], G9P[4], and G9P[7]). The combination G6P[5] predominated in this study (45.5%, 5/11).

**Figure 3:**
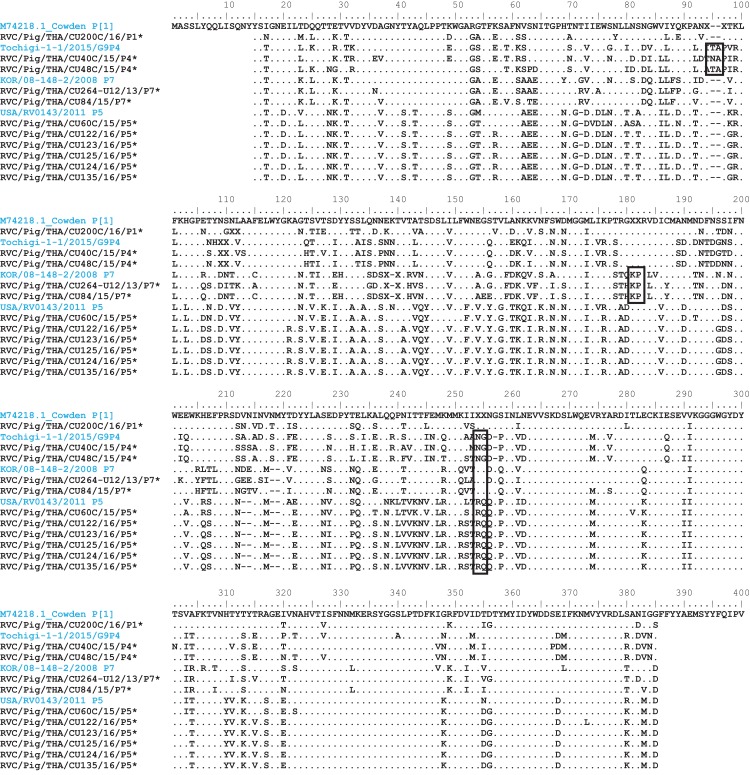
Amino acid alignment of the deduced amino acid residues encoded by the RVC VP4 gene. Residue positions 15–385 were numbered based on the prototype strain Cowden (genotype P[1]). Other reference strains belonging to P[4], P[7], and P[5]. Reference strains are in blue, while strains from this study are noted by asterisks. Dots represent identical residues to the prototypic Cowden. X represents unknown amino acids due to missing nucleotides in the alignment. Deletions are denoted with dash; insertions are boxed.

Analysis of the Thai RVC intra-genotype nt sequences showed between 79.5% (for P[4] strains) and 80.7% (for P[7] strains). Sequence identity for P[5] strains was >99.8%. The deduced amino acid sequences of several representative RVC strains from this study were compared with the amino acid sequences of the prototype strains (Fig. 3). The alignment region spanned residues 15–385 (based on Cowden numbering). Regions of exceptionally high conservation were more frequent towards the carboxyl than the amino terminus, especially in the last one-third of the sequence. Of interest is the two residues deletion at position 111–112 of the RVC/Pig/THA/CU200C/16/P1 compared to the Cowden strain. Other deletions found appeared to be genotype-specific, such as at positions 109–110 for P[4] strains, position 257 for P[4], positions 72, 213, and 214 for P[5], and positions 138–140 for P[7]. Hypervariation such as at positions 228, 236, and 241 were located throughout the sequence.

## Discussion

The viral etiology of diarrhea in pigs is not routinely investigated on Thai pig farms, which often contributes to the failure to prevent and contain disease transmission. Rotavirus infection including RVC disproportionately affects suckling and weaned piglets and often co-infects with other viruses (Saif et al., 1980; Amimo, Vlasova & Saif, 2013; Martella et al., 2007). Previous reports of porcine rotavirus prevalence in Thailand have only been RVA (approximately 10–23%), while epidemiological study of porcine RVC was limited (Chan-It et al., 2008; Khamrin et al., 2007; Maneekarn & Khamrin, 2014; Yodmeeklin et al., 2016). Our passive surveillance identified a lower prevalence of RVC (6.6%) than RVA (9.5%) in symptomatic piglets with diarrhea, both of which were detected as single and/or co-infections (Collins, Martella & O’Shea, 2008; Marthaler et al., 2014; Nagesha & Holmes, 1988; Theuns et al., 2016; Zhou et al., 2016). RVC appeared most frequently in pigs eight weeks old or younger, which was consistent with previous reports (Amimo, Vlasova & Saif, 2013; Jeong et al., 2009; Kim et al., 1999; Martella et al., 2007; Marthaler et al., 2013; Suzuki et al., 2015).

It is believed that animals co-infected with more than one enteric virus experienced increased intestinal epithelium damage and/or viral replication, which results in more severe diarrhea (Amimo, Vlasova & Saif, 2013; Jeong et al., 2009; Ishimaru et al., 1991; Martella et al., 2007). In this study, dual infections between PEDV and rotavirus in younger piglets (<4 weeks old) often showed a higher morbidity rate. Younger piglets appear to be more susceptible to higher morbidity and mortality than older pigs (Annamalai et al., 2015; Shibata et al., 2000; Steyer et al., 2008). There were instances of co-infection with PEDV and RVA in sows even though they are usually asymptomatic. This may explain the persistence of rotavirus within the herd and facilitate vertical transmission. Although rotavirus infection is frequent in the winter season on farms in the temperate climate, rotavirus infection occurred throughout the year on Thai swine farms. Some studies have suggested that rotavirus infection is not as seasonally dependent in the tropics because the relatively high humidity may facilitate increased rotavirus infection (Cook et al., 2004; Levy, Hubbard & Eisenberg, 2009).

Genetic analysis of the VP7 gene from RVC-positive samples showed varying nt sequence identities for G1 (between 83.7% and 100%), G6 (82.2–100%), and G9 (83.2–100%). It was interesting to note that one G6 strain RVC/Pig/THA/CU146C/16/G6 did not cluster with the other strains, which was not surprising given its low sequence identity of 82.2–84.4%. The deduced amino acid sequence of RVC/THA/CU146C/16/G6 lacked four amino acid residues between positions 245 and 248 located in the carboxyl terminus of the VR8 region compared to other G6 strains. These deletions may lend additional genetic diversity to this strain and has been observed previously in an Irish study (Collins, Martella & O’Shea, 2008). It would have been interesting to determine if these residues correlate with any differences in disease severity, but unfortunately no clinical data were made available to us by the farm from which the sample was submitted.

The predominant G1 genotype reported in this study was similar to findings from Ireland, the USA, Canada, and the Czech Republic (Collins, Martella & O’Shea, 2008; Marthaler et al., 2013; Moutelikova, Prodelalova & Dufkova, 2015). Although only one sample belonging to G3 was identified in this study, G3 and G7 were the most frequently detected genotypes elsewhere in Asia (Jeong et al., 2015). Mixed G genotypes within the same farm were occasionally found, such as RVC/Pig/THA/CU14/15/G1 and RVC/Pig/THA/CU13/15/G9 from Farm V, and RVC/Pig/THA/CU275C/16/G9, RVC/Pig/THA/CU276C/16/G9, and RVC/Pig/THA/CU330C/16/G1 from Farm K. Poor management and overcrowded conditions on the farms often lead to co-circulation of multiple enteric pathogens or mixed viral infections (Martella et al., 2007).

The genetic relationship between VP4 sequences in this study and previous RVC isolates was also determined. The sequence identity among the Thai RVC strains and the prototypic strains was quite low. Comparison showed between 59.6% and 66% with the Cowden strain, 52.2% and 62.7% with the human strain Bristol, and 59.5% and 66.1% with the bovine strain Shintoku. Not surprisingly, most strains analyzed in this study possessed nt identities similar to previously reported Asian RVC strains (Korean and Japanese strains) (Jeong et al., 2015; Suzuki et al., 2015). Taken together, these data suggest that the same RVC genotypes are in circulation within several Asian countries.

This study was limited by the inability to successfully sequence all the VP4 gene from all 47 VP7-positive RVC, which suggests possible high sequence variability in the region we targeted for amplification. The fact that P[5] sequences were nearly identical reflected the single origin and time of infection. Indeed, these samples were derived from the same farm, which suggests an RVC outbreak and may not truly represent the variability of all the strains in circulation.

## Conclusion

Rotavirus group C surveillance in Thailand and Southeast Asia will continue to be important in identifying the viral etiology of gastroenteritis in pigs and in managing the viral transmission on swine farms. This study identified several currently circulating RVC in swine, an economically important food animal and a potential source of zoonotic transmission of rotavirus.

## Supplemental Information

10.7717/peerj.4724/supp-1 Supplemental Information 1 Details of the farm location and pig age for which samples were derived.

10.7717/peerj.4724/supp-2 Supplemental Information 2 Multiple sequence alignments of the eight variable regions (VR1–VR8) of RVC.

10.7717/peerj.4724/supp-3 Supplemental Information 3 Age distribution of pig samples tested positive for PEDV, RVA and RVC.

10.7717/peerj.4724/supp-4 Supplemental Information 4 The raw data of RVC detection. Total case submission, RVC positive case and RVC genotype (VP7).

10.7717/peerj.4724/supp-5 Supplemental Information 5 Sequences for RVC VP7.

10.7717/peerj.4724/supp-6 Supplemental Information 6 Sequences for RVC VP4.

## Additional Information and Declarations

### Data Availability

The following information was supplied regarding data availability: Total cases submitted between 2011 and 2016, RVC positive cases and RVC genotypes can be found in the raw data in the Supplemental Files.
