# Supplementary material for: Porcine rotavirus C in pigs with gastroenteritis on Thai swine farms, 2011–2016
Source: PeerJ. 2018 May 8;6:e4724. doi: 10.7717/peerj.4724 (PMC5947060; doi:10.7717/peerj.4724)
Supplement: Supplemental Information 1 [file peerj-06-4724-s001.docx]

**Supplementary Table 1. Details of the farm location and pig age for which samples were derived.**

|  | **2011-2016** | | | | | | | |
| --- | --- | --- | --- | --- | --- | --- | --- | --- |
|  | **Total** | **0-6 d** | **1-4 wk** | **>4-8 wk** | **>8-12 wk** | **>12 wk** | **Sow** | **N/A** |
| **Central** | **173** |  |  |  |  |  |  |  |
| Lop Buri | 2 |  | 1 | 1 |  |  |  |  |
| Samut Songkhram | 3 |  | 3 |  |  |  |  |  |
| Suphan Buri | 32 | 12 | 18 |  |  |  | 1 | 1 |
| Saraburi | 16 | 3 | 4 | 8 |  |  | 1 |  |
| Phranakhon Si Ayutthaya | 6 | 6 |  |  |  |  |  |  |
| Nakhon Pathom | 114 | 36 | 31 | 16 | 2 | 12 | 5 | 12 |
| **Western** | **316** |  |  |  |  |  |  |  |
| Kanchanaburi | 16 | 0 | 8 | 7 |  |  |  | 1 |
| Prachuap Khiri Khan | 4 |  | 4 |  |  |  |  |  |
| Phetchaburi | 2 |  | 2 |  |  |  |  |  |
| Ratchaburi | 294 | 36 | 131 | 89 | 12 | 5 | 11 | 10 |
| **Eastern** | **109** |  |  |  |  |  |  |  |
| Chon Buri | 92 | 27 | 21 | 10 | 0 | 34 |  |  |
| Chachoengsao | **17** | 10 | 1 |  |  | 6 |  |  |
| **Northeastern** | **80** |  |  |  |  |  |  |  |
| Ubon Ratchathani | 4 | 4 |  |  |  |  |  |  |
| Udon Thani | 4 |  | 4 |  |  |  |  |  |
| Nakhon Ratchasima | 72 | 10 | 2 | 9 | 15 | 14 | 22 |  |
| **Southern** | **26** |  |  |  |  |  |  |  |
| Trang | 23 |  | 9 | 6 |  |  |  | 8 |
| Nakhon Si Thammarat | 3 |  | 3 |  |  |  |  |  |
| **Unspecified location** | 65 | 3 | 2 | 3 |  | 1 | 1 | 55 |

N/A, not available.
