# Supplementary figures and images for: Porcine rotavirus C in pigs with gastroenteritis on Thai swine farms, 2011–2016
Source: PeerJ. 2018 May 8;6:e4724. doi: 10.7717/peerj.4724 (PMC5947060; doi:10.7717/peerj.4724)

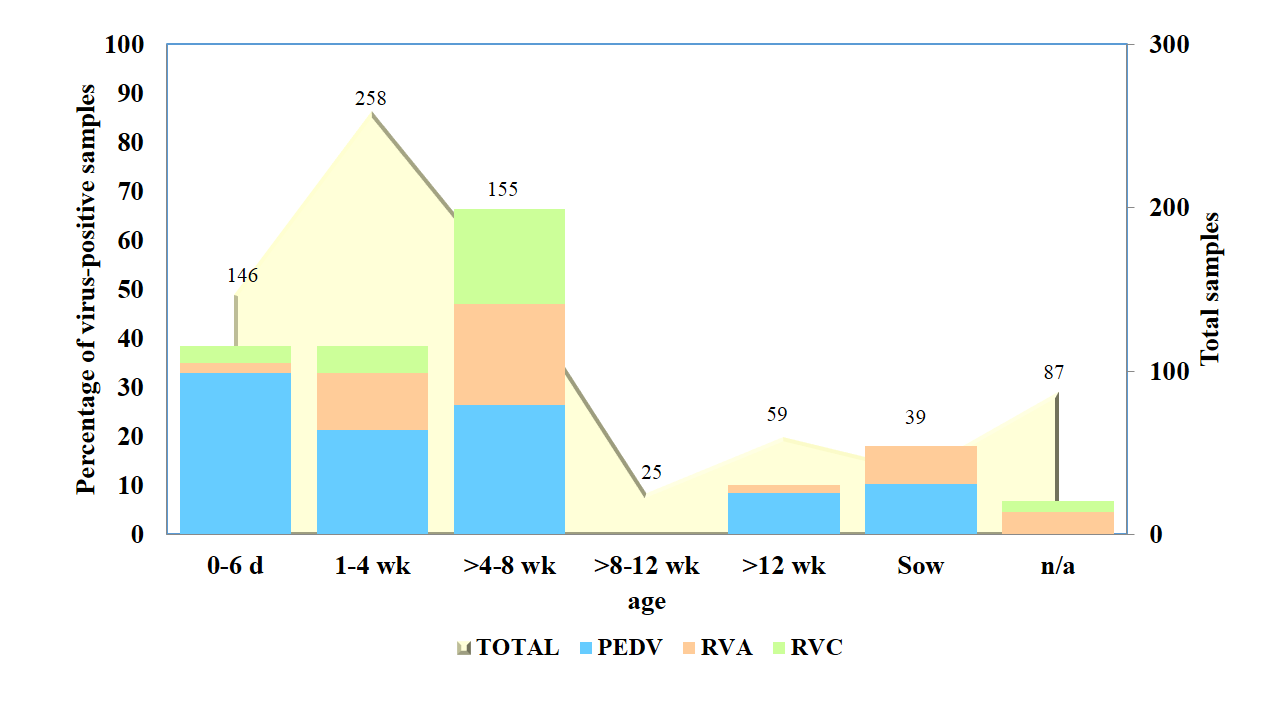

Supplement: Supplemental Information 3 [file peerj-06-4724-s003.tif]
